# Plant-based diet, metabolic signature, genetic susceptibility, and risk of musculoskeletal disorders: a large-scale population-based prospective cohort study

**DOI:** 10.3389/fpubh.2026.1803631

**Published:** 2026-06-29

**Authors:** Sirui Zheng, Xin Song, Bin Yang, Minghao Jin, Xiaofeng Ma, Di Zhang, Bowen Lei, Yang Qu, Rong Xiang, Xunying Zhao, Tao Han, Jinyu Zhou, Ting Yu, Maoyao Xia, Yangdan Zhong, Jiawei Liu, Jiayuan Li, Mengyu Fan, Xia Jiang

**Affiliations:** 1 Department of Nutrition and Food Hygiene, West China School of Public Health and West China Fourth Hospital, Sichuan University, Chengdu, Sichuan, China; 2 Department of Epidemiology and Biostatistics, West China School of Public Health and West China Fourth Hospital, Sichuan University, Chengdu, Sichuan, China; 3 Department of Clinical Neuroscience, Karolinska Institute, Stockholm, Sweden

**Keywords:** cohort study, genetic susceptibility, metabolic signature, musculoskeletal disorders, plant-based diet

## Abstract

**Aims:**

To explore the association between plant-based diets, diet-related metabolic signatures, and the risk of total and specific musculoskeletal disorders (MSDs), while accounting for genetic susceptibility.

**Methods:**

We analyzed data from 107,983 participants in the UK Biobank. Plant-based diet indices [PDI, unhealthy PDI (uPDI), and healthy PDI (hPDI)] were calculated via 24-h dietary recalls. Elastic net regression was applied to generate PDIs-related metabolic signatures. Polygenic risk score (PRS) was constructed to quantify genetic susceptibility to MSDs. Cox proportional hazards regression models were used to evaluate the associations of PDIs, metabolic signatures, and PRS with the risk of incident MSDs. Mediation analysis was conducted to assess whether metabolic signatures mediated the PDIs–MSDs relationship.

**Results:**

Over a median follow-up of 11.6 years, 31,097 participants developed any MSDs. Higher PDI was associated with a reduced risk of total MSDs (HR = 0.98, 95% CIs = 0.96–1.00) whereas higher uPDI was associated with an increased risk of total MSDs (HR = 1.02, 95% CIs = 1.00–1.04). The magnitude of effect became more pronounced when using the corresponding PDI-related metabolic signature as exposure (HR = 0.97, 95% CIs = 0.95–0.98). While no significant effect was found for hPDI (HR = 1.00, 95% CIs = 0.98–1.02), its metabolic signature was associated with a lower risk of total MSDs (HR = 0.98, 95% CIs = 0.96–1.00). Results remained consistent across different genetic risk strata. No significant mediating effects of metabolic signatures on the PDIs-MSDs associations were observed. When extended to the five specific MSDs (rheumatoid arthritis, osteoarthritis, lower back pain, neck pain and gout), similar patterns of results were observed.

**Conclusions:**

Adherence to a healthy plant-based diet as reflected by higher PDI and hPDI is associated with a lower risk of total and specific MSDs, while an unhealthy plant-based diet increases is associated with an elevated risk of these conditions. Our findings support the potential of a healthy plant-based diet in maintaining musculoskeletal health as well as the utility of metabolic signatures for optimizing dietary assessment and guiding personalized dietary interventions.

## Introduction

Musculoskeletal disorders (MSDs) encompassing rheumatoid arthritis (RA), osteoarthritis (OA), low back pain (LBP), neck pain (NP) and gout, pose a significant public health concern (1). Characterized by debilitating symptoms such as chronic pain, limited mobility and functional impairment, MSDs contribute to high rates of incidence and disability worldwide (2). According to the Global Burden of Disease study, MSDs affected over 1.68 billion individuals and accounted for 161.88 million disability-adjusted life years in 2021 (3). The fact that MSDs compromise physical, psychological and functional status, threaten healthy aging, and contribute to premature mortality, makes the identification of modifiable risk factors critical (4, 5).

Plant-based diets has gained increasing attention over the past years for both its health benefits (6–11) and environmental sustainability (12). Despite a growing interest in understanding the roles of plant-based diets in MSDs, existing studies have narrowly focused on specific conditions like RA (13, 14) or gout (15), neglecting the broader spectrum of common MSDs. These studies also suffer from small sample sizes and a lack of quantitative dietary assessments, preventing a precise measurement of how different dietary compositions influence disease risk. A comprehensive, large-scale prospective cohort study is therefore needed to systematically explore the relationship between plant-based diets and MSDs and to elucidate the underlying biology.

Plant-based diets modulates metabolic pathways underlying inflammation, oxidative stress, and nutrient metabolism, all of which are key processes in MSDs pathogenesis and may act as critical mediators bridging dietary patterns influence the development of disease (16). Unfortunately, conventional studies typically focus on the mediatory roles of isolated biomarkers such as C-reactive protein (17) or plasma triglycerides (TGs) (18), failing to capture the complexity of the overall metabolic network. Moreover, individual variability often leads to divergent metabolic responses even when exposed to the same diet, a nuance that cannot be captured by examining only a handful of specific metabolites. High-throughput metabolomics addresses these limitations by quantifying thousands of circulating metabolites, thereby characterizing the dynamic dietary-induced metabolic remodeling (19). This method offers a powerful opportunity to objectively assess the metabolic responses to plant-based diets and to determine how these changes influence the subsequent onset of MSDs, thereby advancing our mechanistic understanding as well as identifying potential interventional targets.

The development of MSDs is driven by interactions between environmental triggers and genetic susceptibility (20). Genome-wide association studies (GWAS) have successfully identified single nucleotide polymorphisms (SNPs) associated with specific MSDs (21–23), laying a foundation for evaluating genetic risk via polygenic risk score (PRS). Recent developments, such as the metaPRS, combine multiple trait-specific PRSs into a single score to improve predictive accuracy for complex diseases (24). Despite this, no studies have yet investigated the interactions between plant-based diets and genetic susceptibility in relation to MSDs risk.

Therefore, this study aims to (1) longitudinally investigate the association between plant-based diets and the risk of overall MSDs; (2) identify metabolic signatures reflecting plant-based diets and assess their potential mediating roles; and (3) investigate whether the associations of plant-based diets and their related metabolic signatures with MSDs risks differ across the genetic susceptibility stratum. Additionally, we extend these analyses to five specific MSDs. By integrating these multi-omics dimensions within a population-based prospective cohort, we seek to advance the mechanistic understanding of MSDs and inform personalized dietary strategies for their prevention.

## Methods

### Study population

We used data from the UK Biobank (UKB), a large-scale prospective cohort study comprising over 500,000 participants aged 40–69 years, who were recruited between 2006 and 2010. Participants completed a touch-screen questionnaire, a face-to-face interview, a series of physical measurements, and provided biological samples (25). The UKB study received ethical approval from the NHS North-West Multi-Center Research Ethics Committee (Ref. 11/NW/0382), and every participant provided written informed consent.

We included participants who completed at least one 24-h dietary questionnaire (*n* = 210,944) and excluded those with implausible body mass index (BMI; <15 or >50 kg/m^2^, *n* = 843), extreme energy intake (<500 or >3,500 kcal/day in females; <800 or >4,200 kcal/day in males, *n* = 2,594), non-European ancestry (*n* = 9,204), missing genetic data (*n* = 3,642) and those with prevalent MSDs at baseline. For the epidemiological analysis targeting total MSDs, 107,983 individuals were included. For the metabolic analysis, we additionally excluded participants with incomplete metabolomic data, resulting in a final sample size of 52,562 participants. The sample sizes for the epidemiological analysis of specific MSDs including RA, OA, LBP, NP and gout were 192,434, 166,575, 175,885, 194,278 and 190,445, respectively; and for the metabolic analysis were 95,478, 82,029, 87,010, 96,447 and 94,452, respectively. Data selection process is illustrated in Supplementary Figure 1.

### Dietary assessment

Dietary data in UKB were collected using the Oxford WebQ, based on 24-h dietary recall questionnaire covering more than 200 food items and over 30 beverage types, conducted up to five cycles during 2009–2012 (26). Participants who completed at least one 24-h dietary assessment were included. The distribution of valid recall cycles was 40,600 (37.60%) with one cycle only, 24,911 (23.07%) with two, 22,737 (21.06%) with three, 16,533 (15.31%) with four and 3,202 (2.97%) with five cycles. For those who completed two or more assessments, the average intake of each food item was computed based on responses across all valid assessments.

As the primary exposure in the present study, plant-based diet indices (PDIs), including the overall plant-based diet index (PDI), healthy plant-based diet index (hPDI), and unhealthy plant-based diet index (uPDI), were calculated following the established methodology proposed by Satija et al. (8, 9). Foods were first categorized into 17 distinct groups further grouped into three categories: healthy plant-based foods, less healthy plant-based foods, and animal foods (Supplementary Table 1). Intake of each of the 17 food groups was ranked into quintiles and was assigned either positive scores (Q1–Q5 received 1–5 point) or reverse scores (Q1–Q5 received 5–1 point). For the PDI, positive scores were assigned to both healthy and less healthy plant-based food groups, while reverse scores were assigned to animal food groups. For hPDI, positive scores were restricted to healthy plant-based food groups, while reverse scores were applied to both less healthy plant-based food groups and animal food groups. For uPDI, positive scores were assigned to less healthy plant-based food groups, while reverse scores were assigned to both healthy plant-based food groups and animal food groups. The PDIs for an individual were the sum of scores across all 17 food groups, with a theoretical range of 17–85.

### Measurement of metabolic biomarkers

Metabolic profiling utilizing Nuclear magnetic resonance (NMR) metabolic biomarker data generated by Nightingale Health were derived from EDTA-treated samples of ~280,000 participants in UKB (27). A subset of 16,000 participants completed a repeated assessment. Initial analyses of metabolites were conducted in Finland using advanced spectrometry techniques, incorporating a comprehensive profiling process and rigorous quality control protocols as described previously (28). A total of 251 biomarkers were identified, containing 170 directly measured biomarkers and 81 metabolite ratios, including glycoprotein acetyls, lipoprotein lipids in 14 subclasses, fatty acids (FAs), amino acids, ketone bodies and glycolysis metabolites (29). These circulating metabolic biomarkers were further used to construct diet-related metabolic signatures serving as potential biological mediators linking plant-based dietary patterns to MSDs.

### Assessment of outcomes

The primary outcome of this study was total MSDs. Secondary outcomes comprised five specific MSDs (RA, OA, LBP, NP, and gout). Identification of MSDs relied on any hospital admission records coded using the International Classification of Diseases, Ninth Revision (ICD-9) or Tenth Revision (ICD-10). Details on the ICD-9 and ICD-10 codes are shown in Supplementary Table 2. For this study, the baseline time point was defined as the date when participants first completed their 24-h dietary recall questionnaire. Follow-up duration was calculated from baseline to the earliest of the following events: diagnosis of a MSD, death, loss to follow-up, or end of follow-up.

### Measurement of polygenic risk score

Genetic susceptibility to MSDs was quantified as an effect modifier to explore genetic heterogeneity in diet-metabolism-disease pathways. The PRS for RA was obtained from “Standard PRS” provided in the UK Biobank Release, whose predictive performance has been validated (30). For the other four specific MSDs, PRSs were calculated using PLINK. Details regarding genotyping procedures, quality control protocols, imputation datasets and additional specifics of the UKB genetic data have been published previously (31). To construct PRSs for OA, LBP, NP, and gout, we first extracted SNPs significantly associated with the risk of each disease, which were identified from well-designed, previously published GWAS. SNPs were then filtered to exclude those with a minor allele frequency (MAF) <0.005 and an imputation quality score <0.4. Supplementary Tables 3–5 provide information on the candidate SNPs utilized in calculating PRSs. To quantify the genetic susceptibility to total MSDs, a metaPRS was constructed integrating trait-specific PRSs. Each individual PRS was standardized to zero mean and unit standard derivation (SD). Subsequently, an elastic-net penalized Cox regression analysis was performed using the R package “glmnet” (39) to examine associations between the trait-specific PRSs and total MSDs risk, adjusting for age, sex, genetic arrays, and the top 10 principal component. A 10-fold cross-validation was employed to evaluate multiple models with varying penalty parameters, facilitating the identification of the optimal model. The model yielding the highest cross-validated area under the receiver operating characteristic curve (AUC) was selected as the final model, and the adjusted coefficients for each PRS were extracted as weights. The metaPRS was ultimately computed as a weighted sum (Equation 1):


$$\begin{array}{l} metaPRS_{i}=\frac{\alpha_{1}PRS_{i1}+\alpha_{2}PRS_{i2}+\ldots {+\alpha }_{5}PRS_{i5}}{\alpha_{1}+\ldots +\alpha_{5}} \end{array}$$ (1)

where *PRS*_*i*1_,…, and *PRS*_*i*5_ are the five standardized trait-specific PRSs corresponding to the ith individual; α_1_,…, and α_5_ are the coefficients for each of the PRSs.

Based on the distribution of either trait-specific PRSs or metaPRS among participants, individuals were divided into low (below or equal to the median), or high (above the median) genetic risk categories.

### Covariates and definition of lifestyle risk categories

All covariates were obtained from the UK Biobank standardized assessments, including touch-screen questionnaires, face-to-face interviews, and objective physical measurements. Covariates comprised age (continuous), sex (male/female), education level (high education/ low education), Townsend Deprivation Index (TDI, Q1–Q3), lifestyle risk (low or high, defined in detail below), energy intake (continuous; estimated from the 24-h dietary recall data), multivitamin use (yes/no), and medication use (antihyperlipidemic, antihypertensive, and antidiabetic medications, each documented as yes or no).

Based on prior studies on lifestyle factors and guidelines (32–38), five key lifestyle factors were selected to determine a participant's health behavior status: body mass index (BMI), physical activity, smoking status, alcohol consumption, and sleep duration. A “healthy behavior” was defined for each factor as follows: BMI ≤ 25 kg/m^2^, moderate-to-high physical activity level, never-smoking, never-drinking, and sleep duration ≥7 h/day. Each factor was assigned a binary score (0 for healthy behavior, 1 for unhealthy behavior), and the total lifestyle factor score was calculated as the sum (range from 0 to 5). Participants were further divided into low (total score ≤ 2) and high (total score >2) “conventional lifestyle risk” categories.

### Statistical analysis

All statistical analyses were performed using R software (version 4.3.0, R Foundation for Statistical Computing, Vienna, Austria), with two-sided *P* < 0.05 considered statistically significant. Continuous variables were summarized as mean ± SD, while categorical variables were presented as frequency (percentage). Missing values in covariates were addressed using multivariate imputation by chained equations (MICE). To compare characteristics between participants with MSDs and the control group without MSDs, *t*-tests were used for continuous variables and chi-square tests were applied for categorical variables. To provide clearer characterization of dietary intake and validate the classification performance of the PDIs, multivariable linear regression models were used to estimate adjusted mean differences in scores of 17 component food group between the highest and lowest quintiles of each PDIs.

PDIs-related metabolic signatures were constructed based on identified PDIs-associated metabolites through three key steps. First, the 251 metabolites underwent log-transformation followed by *z*-score normalization to ensure consistent scaling. Second, multivariable-adjusted linear regression was used to estimate associations between each PDI and individual metabolites, with Bonferroni-corrected *P* < 0.05 set as threshold for significance. Third, metabolites with significant associations were included in the elastic net regression, a regularized linear method that combines Lasso and Ridge penalties (39). This approach was chosen as it not only enhances robustness against the multicollinearity common in high-dimensional metabolomic data, but also retains variable selection functionality while reducing overfitting, making it ideal for constructing stable and reliable diet-related metabolic signatures. The alpha and lambda parameters in the regression were optimized via a 10-fold cross-validation, with the minimum mean squared error used as the selection criterion (40). We calculated the PDIs-related metabolic signature as the weighted sum of selected metabolites, with weights corresponding to the regression coefficients from the elastic net model. To validate the performance of the established metabolic signature, parameters from the trained model were applied to the first repeated assessment dataset. The metabolic signature was further stratified into unfavorable (below or equal to the median), or favorable (above the median) categories.

Cox proportional hazards models were used to estimate the hazard ratios (HRs) with 95% confidence intervals (95% CIs) for the associations of PDIs, metabolic signatures, and individual metabolites with the risk of MSDs. The PDIs and metabolic signatures were analyzed both as quintile-based categorical variables and as continuous variables. The proportional hazards assumption was verified using Schoenfeld residuals and no violation was detected (Supplementary Table 11). To address potential confounding, distinct hierarchical adjustment models were established for different exposure-outcome associations. For the associations between PDIs and MSDs, three models were used: model 1 was adjusted only for age and sex; model 2 was further adjusted for TDI, education, lifestyle factors, energy intake, and multivitamin use; model 3 was built on top of model 2 with additional adjustment for medication use. For the associations between metabolic signatures and MSDs, model 1 was adjusted only for age and sex; model 2 was further adjusted for TDI, education, lifestyle factors, energy intake, multivitamin use, and medication use; model 3 was built on top of model 2 with additional mutual adjustment for PDIs and metabolic signatures to evaluate their independent associations with MSDs risk.

Mediation analyses were additionally performed using the R package “CMAverse” [(41); https://github.com/BS1125/CMAverse.git] to explore whether metabolic signatures or individual metabolites mediated the PDIs-MSDs associations. Linear regression models were employed to characterize the associations between the exposure and the mediator. Subsequently, Cox proportional hazards models were utilized to analyze the association between the mediator and the outcome. By integrating these two models, we estimated the indirect, direct, and total effects. Additionally, 95% CIs for the proportion mediated were calculated using nonparametric bootstrap resampling. Candidate mediators were selected based on significant associations with both the exposure and outcome within the hypothesized causal pathway.

We further examined the joint association of PDIs, metabolic signatures, and conventional lifestyle risk with MSDs, with the reference group defined as the lowest-risk subgroup (e.g., those with a high PDI or favorable metabolic signature and a low conventional lifestyle risk). Stratified analyses were also conducted across conventional lifestyle risk strata to assess the PDIs/metabolic signatures-MSDs associations within each stratum. To quantify interactions, additive interactions were evaluated using the relative excess risk due to interaction (RERI), attributable proportion due to interaction (AP), and synergy index with 95% CIs, while multiplicative interactions were tested via likelihood ratio tests by comparing models with and without an interaction term. The same framework was applied to investigate combined effects of PDIs, metabolic signatures, and PRS on MSDs risk.

Sensitivity analyses were conducted to confirm the robustness of results. First, participants diagnosed with MSDs within the initial 1 year of the follow-up period were excluded to mitigate potential reverse causality. Second, the analysis was limited to participants with at least two dietary assessments. Third, Fine and Gray's sub-distribution hazard models were employed to address the competing risk of death. Finally, we explored the associations between each of the 17 PDIs component food groups and MSDs risk.

## Results

### Characteristics of the study participants

The baseline characteristics of participants involved in the study of total MSDs are presented in Table 1 and of each specific MSDs are provided in Supplementary Tables 6–10. Generally, the MSDs group had a lower average PDI, was older, had more females, was less well-educated, had a higher average BMI, exercised more, had more previous and current smokers, had fewer current alcohol drinkers, had lower energy intake, used fewer multivitamins but more medications.

**Table 1:** Baseline characteristics of participants by total MSDs incidence.

Characteristic	All	Incident total MSDs		*P*-value
		Yes	No	
Participants, *n*	107,983^*^	31,097	76,886	
Plant-based diet index				
PDI	54.13 (5.44)	54.02 (5.44)	54.18 (5.43)	**<0.001**
hPDI	57.78 (5.81)	57.81 (5.80)	57.76 (5.81)	0.226
uPDI	56.31 (5.78)	56.30 (5.78)	56.32 (5.78)	0.499
Age, years	57.42 (8.10)	59.12 (7.85)	56.74 (8.10)	**<0.001**
Sex, male	49, 992 (46.30%)	13,747 (44.21%)	36,245 (47.14%)	**<0.001**
TDI				
Q1	39,230 (36.37%)	11,151 (35.89%)	28,079 (36.56%)	0.117
Q2	37,874 (35.11%)	10,990 (35.37%)	26,884 (35.01%)	
Q3	30,764 (28.52%)	8,928 (28.74%)	21,836 (28.43%)	
Education				
High education level	64,841 (60.25%)	17,586 (56.83%)	47,255 (61.64%)	**<0.001**
Less than college or university degree	42,772 (39.75%)	13,360 (43.17%)	29,412 (38.36%)	
Lifestyle characteristics				
BMI, kg/m^2^				
<25	44,191 (40.92%)	11,453 (36.83%)	32,738 (42.58%)	**<0.001**
25–30	44,551 (41.26%)	12,999 (41.80%)	31,552 (41.04%)	
≥30	19,241 (17.82%)	6,645 (21.37%)	12,596 (16.38%)	
Physical activity				
Low	16,131 (17.80%)	4,459 (17.47%)	11,672 (17.93%)	**<0.001**
Moderate	38,959 (42.99%)	10,752 (42.12%)	28,207 (43.33%)	
High	35,530 (39.21%)	10,315 (40.41%)	25,215 (38.74%)	
Smoking status				
Never	62,578 (58.06%)	16,871 (54.36%)	45,707 (59.55%)	**<0.001**
Previous	36,844 (34.18%)	11,680 (37.64%)	25,164 (32.79%)	
Current	8,361 (7.76%)	2,482 (8.00%)	5,879 (7.66%)	
Alcohol consumption				
Never	2,553 (2.37%)	771 (2.48%)	1,782 (2.32%)	**0.002**
Previous	2,760 (2.56%)	869 (2.80%)	1,891 (2.46%)	
Current	102,632 (95.08%)	29,440 (94.72%)	73,192 (95.22%)	
Sleep duration, h				
<7	22,372 (20.72%)	6,997 (22.50%)	15,375 (20.00%)	**<0.001**
7–8	79,206 (73.35%)	22,100 (71.07%)	57,106 (74.27%)	
>8	6,405 (5.93%)	2,000 (6.43%)	4,405 (5.73%)	
Energy, kcal/day	2,061.15 (543.28)	2,051.54 (548.56)	2,065.04 (541.08)	**<0.001**
Multivitamin supplement	19,704 (18.25%)	5,829 (18.74%)	13,875 (18.05%)	**0.007**
Medication use				
Antihyperlipidemic	13,956 (12.92%)	5,194 (16.70%)	8,762 (11.40%)	**<0.001**
Antihypertensive	16,799 (15.56%)	6,240 (20.07%)	10,559 (13.73%)	**<0.001**
Antidiabetic	687 (0.64%)	295 (0.95%)	392 (0.51%)	**<0.001**

^*^Please note 107,983 is the number of participants with total MSDs. Specifically, the numbers of participants with RA, OA, low back pain, neck pain and gout are 192,434; 166,575; 175,885; 194,278 and 190,445, respectively. Data were expressed as mean (standard deviation) or n%, and P value were determined by Student t test or Wilcoxon rank-sum test. Bold values indicate statistically significant results (*P* < 0.05). MSDs, musculoskeletal disorders; PDI, plant-based diet index; hPDI, healthy plant-based diet index; uPDI, unhealthy plant-based diet index; TDI, Townsend deprivation index; BMI, body mass index; RA, rheumatoid arthritis; OA, osteoarthritis.

As shown in Supplementary Table 12, fruit juices were the primary driver of the overall PDI, with a significantly higher score in the highest vs. lowest quintile. For the hPDI, refined grains represented the most distinguishing food group, with the most pronounced reduction in adjusted scores in the highest quintile. For the uPDI, legumes were the key differentiating food group, but with a markedly lower adjusted score in the highest quintile.

### Associations of PDIs with the risk of MSDs

Higher PDI was significantly associated with a decreased risk of total MSDs in the minimally adjusted model (Table 2). Compared with individuals in the lowest quintile (Q1), individuals in the highest quintile (Q5) had a significantly reduced risk of total MSDs (HR = 0.95, 95% CIs = 0.92–0.99); each 10-unit increment in PDI was also associated with a 3% reduced risk (HR = 0.97, 95% CIs = 0.95–0.99). These associations remained directionally consistent after adjusting for multiple covariates, despite a slight attenuation of significance. For specific MSDs, higher PDI was associated with a lower risk of OA (HR = 0.96, 95% CIs = 0.94–0.98) and gout (HR = 0.93, 95% CIs = 0.87–0.99) while no significant association was observed for RA, LBP or NP (Supplementary Table 13).

**Table 2:** HRs and 95% CIs for total musculoskeletal disorders, according to PDIs and metabolic signature among participants from the UK Biobank.

PDIs	HR (95% CI)^a^			Metabolic signature of PDIs	HR (95% CI)^b^		
	Model 1	Model 2	Model 3		Model 1	Model 2	Model 3
PDI				Metabolic signature of PDI			
Q1	Reference	Reference	Reference	Q1	Reference	Reference	Reference
Q2	**0.97 (0.94**–1.00)^*^	0.98 (0.95–1.01)	0.98 (0.95–1.01)	Q2	**0.93 (0.88**–0.98)^**^	**0.95 (0.90**–1.00)^*^	**0.95 (0.90**–1.00)^*^
Q3	0.96 (0.93–1.00)	0.98 (0.94–1.02)	0.98 (0.94–1.02)	Q3	**0.91 (0.87**–0.95)^***^	**0.95 (0.90**–1.00)^*^	**0.95 (0.90**–1.00)^*^
Q4	**0.94 (0.91**–0.97)^***^	**0.96 (0.92**–0.99)^**^	**0.95 (0.92**–0.99)^**^	Q4	**0.87 (0.82**–0.91)^***^	**0.92 (0.87**–0.96)^***^	**0.92 (0.87**–0.97)^***^
Q5	**0.95 (0.92**–0.99)^**^	0.98 (0.94–1.01)	0.97 (0.94–1.01)	Q5	**0.85 (0.81**–0.89)^***^	**0.92 (0.88**–0.97)^**^	**0.92 (0.88**–0.97)^**^
Per 10-unit increment	**0.97 (0.95**–0.99)^**^	0.98 (0.96–1.00)	0.98 (0.96–1.00)	Per SD increment	**0.94 (0.92**–0.95)^***^	**0.97 (0.95**–0.98)^***^	**0.97 (0.95**–0.98)^***^
uPDI				Metabolic signature of uPDI			
Q1	Reference	Reference	Reference	Q1	Reference	Reference	Reference
Q2	1.01 (0.97–1.04)	1.00 (0.97–1.04)	1.00 (0.97–1.04)	Q2	1.02 (0.97–1.07)	0.97 (0.92–1.02)	0.97 (0.92–1.02)
Q3	1.02 (0.99–1.05)	1.01 (0.98–1.04)	1.01 (0.97–1.04)	Q3	**1.09 (1.03**–1.14)^**^	1.00 (0.95–1.05)	1.00 (0.95–1.05)
Q4	**1.07 (1.03**–1.10)^***^	**1.05 (1.01**–1.08)^*^	**1.04 (1.01**–1.08)^*^	Q4	**1.15 (1.09**–1.21)^***^	1.01 (0.96–1.07)	1.01 (0.96–1.07)
Q5	**1.07 (1.03**–1.11)^***^	1.03 (1.00–1.07)	1.03 (0.99–1.07)	Q5	**1.22 (1.15**–1.28)^***^	1.02 (0.96–1.08)	1.02 (0.96–1.08)
Per 10-unit increment	**1.05 (1.03**–1.07)^***^	**1.03 (1.01**–1.05)^*^	**1.02 (1.00**–1.04)^*^	Per SD increment	**1.07 (1.05**–1.09)^***^	1.00 (0.99–1.02)	1.00 (0.98–1.02)
hPDI				Metabolic signature of hPDI			
Q1	Reference	Reference	Reference	Q1	Reference	Reference	Reference
Q2	0.98 (0.95–1.02)	0.99 (0.95–1.03)	0.99 (0.95–1.03)	Q2	**0.93 (0.89**–0.98)^**^	0.97 (0.93–1.02)	0.97 (0.93–1.02)
Q3	0.99 (0.95–1.02)	0.99 (0.96–1.03)	0.99 (0.95–1.03)	Q3	**0.90 (0.86**–0.95)^***^	0.98 (0.93–1.03)	0.98 (0.93–1.03)
Q4	1.00 (0.96–1.04)	1.01 (0.97–1.04)	1.00 (0.97–1.04)	Q4	**0.85 (0.81**–0.90)^***^	**0.94 (0.90**–**1.00)** ^*^	**0.94 (0.90**–0.99)^*^
Q5	1.00 (0.96–1.03)	1.01 (0.97–1.04)	1.00 (0.97–1.04)	Q5	**0.84 (0.80**–0.88)^***^	0.95 (0.90–1.01)	0.95 (0.90–1.00)
Per 10-unit increment	1.00 (0.98–1.02)	1.00 (0.99–1.02)	1.00 (0.98–1.02)	Per SD increment	**0.94 (0.92**–0.95)^***^	**0.98 (0.96**–**1.00)** ^*^	**0.98 (0.96**–1.00)^*^

^a^Model 1: Cox regression model adjusted for sex and age. Model 2: Model 1 + TDI, education, physical activity, BMI, smoking status, alcohol consumption, sleep duration, energy intake and multivitamin use. Model 3: Model 2 + history of treatments (antihyperlipidemic drugs, antihypertensive and antidiabetic drugs).

^b^Model 1: Cox regression model adjusted for sex and age. Model 2: Model 1 + TDI, education, physical activity, BMI, smoking status, alcohol consumption, sleep duration, energy intake, multivitamin use and history of treatments (antihyperlipidemic drugs, antihypertensive and antidiabetic drugs). Model 3: Included both PDIs and the metabolic signature simultaneously in the model 2 to examine association independence.

Bold values indicate statistically significant results (*P* < 0.05). The significance thresholds were set at ^*^P < 0.05, ^**^P < 0.01, and ^***^P < 0.001.

PDI, plant-based diet index; uPDI, unhealthy plant-based diet index; hPDI, healthy plant-based diet index; HR, hazard ratio; 95% CI, 95% confidence interval; TDI, Townsend deprivation index; BMI, body mass index.

On the contrary, higher uPDI was significantly associated with an increased risk of total MSDs (Q5 vs. Q1: HR = 1.03, 95% CIs = 0.99–1.07; per 10-unit: HR = 1.02, 95% CIs = 1.00–1.04). For specific MSDs, higher uPDI was associated with an elevated risk of RA (HR = 1.15, 95% CIs = 1.06–1.24), OA (HR = 1.07, 95% CIs = 1.05–1.10), LBP (HR = 1.11, 95% CIs = 1.07–1.14) and NP (HR = 1.29, 95% CIs = 1.15–1.44) except gout. Unfortunately, hPDI was not significantly associated with the risk of either total MSDs or any specific MSDs (Table 2 and Supplementary Table 13).

### Identification of the PDIs-related metabolic signatures

**Figure 1:**
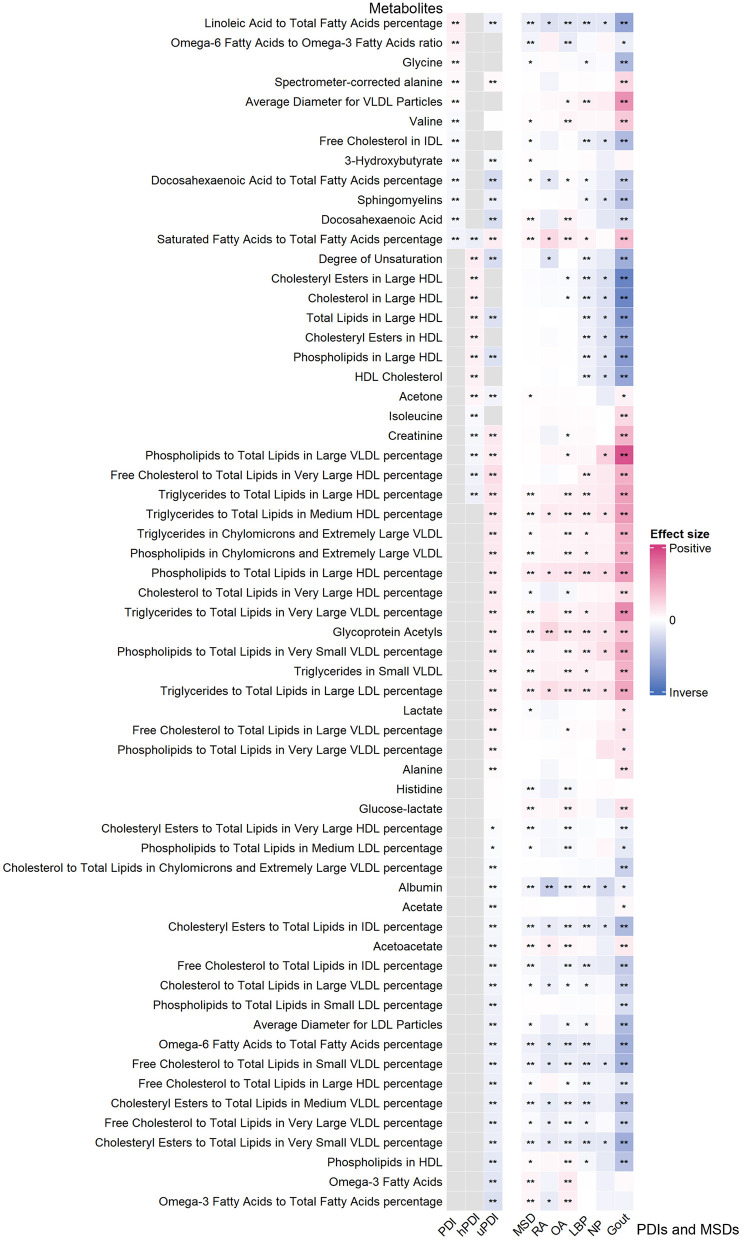
Associations of the 60 metabolites constituting the metabolic signature with plant-based diet indexes and subsequent risk of musculoskeletal disorders. PDI, plant-based diet index; hPDI, healthy plant-based diet index; uPDI, unhealthy plant-based diet index; MSD, musculoskeletal disorder; RA, rheumatoid arthritis; OA, osteoarthritis; LBP, low back pain; NP, neck pain.

Multivariable-adjusted linear regressions identified 91 metabolites significantly (Bonferroni correction) associated with PDI at baseline. These metabolites covered multiple biological pathways, with the largest proportion in lipoprotein subclasses (*n* = 35), followed by FAs (*n* = 13), amino acids (*n* = 10), and relative lipoprotein lipid concentrations (*n* = 5). Elastic net regressions retained 12 metabolites for constructing the PDI-related metabolic signature, including FAs (*n* = 5), amino acids (*n* = 3) and others (*n* = 4). The metabolite signature showed significant correlations with PDI in both the baseline dataset (*r* = 0.08, *P* < 0.001) and the first repeated assessment (*r* = 0.09, *P* < 0.001; Supplementary Figure 2A). For this metabolic signature, the most pronounced contribution to positive coefficients was the percentage of linoleic acid (LA) relative to total FAs, while the most pronounced contribution to negative coefficients was the saturated fatty acids (SFAs) relative to total FAs (Figure 1).

For uPDI, multivariable-adjusted linear regressions identified 205 significant metabolites, of which elastic net regression retained 51 metabolites for constructing the uPDI-related metabolic signature, including relative lipoprotein lipid concentrations (*n* = 23), FAs (*n* = 8), lipoprotein subclasses (*n* = 5), amino acids (*n* = 4), ketone bodies (*n* = 4) and others (*n* = 7). The signature showed significant correlations with uPDI (baseline dataset *r* = 0.20, *P* < 0.001; the first repeated assessment *r* = 0.17, *P* < 0.001; Supplementary Figure 2B). Key contributor to the positive coefficients of uPDI-related signature was the free cholesterol to total lipids in very large high-density lipoprotein (HDL) percentage. In contrast, the percentage of docosahexaenoic acid (DHA) relative to total FAs was main contributor to the negative coefficients (Figure 1).

For hPDI, multivariable-adjusted linear regression identified 184 significant metabolites of which elastic net regression retained 14 metabolites for constructing the hPDI-related metabolic signature, including lipoprotein subclasses (*n* = 4), relative lipoprotein lipid concentrations (*n* = 3), FAs (*n* = 2) and others (*n* = 5). The signature showed significant correlations with hPDI (baseline dataset *r* = 0.094, *P* < 0.001; the first repeated assessment *r* = 0.106, *P* < 0.001; Supplementary Figure 2C). The most prominent contributor to positive coefficients of the hPDI-related signature were the degree of unsaturation of FAs. Conversely, the percentage of SFAs relative to total FAs was the main contributor to negative coefficients (Figure 1).

### Associations of PDIs-related metabolic signatures with the risk of MSDs

Consistent with results of questionnaire-based PDI, the PDI-related metabolic signature showed a robust and even stronger inverse association with total MSDs, where individuals in Q5 had an 8% lower risk than those in Q1 (HR = 0.92, 95% CIs = 0.88–0.97; per-SD: HR = 0.97, 95% CIs = 0.95–0.98; Table 2). Regarding specific MSDs, the PDI-related metabolic signature was associated with a lower risk of OA (HR = 0.97, 95% CIs = 0.95–0.99) and gout (HR = 0.82, 95% CIs = 0.77–0.86) but not on RA, LBP or NP (Supplementary Table 14).

On the other hand, the uPDI-related metabolic signature showed a significant association with the risk of total MSDs in Model 1 (Q5 vs. Q1: HR = 1.22, 95% CIs = 1.15–1.28; per-SD: HR = 1.07, 95% CIs = 1.05–1.09) which remained directionally consistent in multivariable-adjusted models (Table 2). Regarding specific MSDs, the uPDI-related metabolic signature was associated with an increased risk of RA (HR = 1.19, 95% CIs = 1.10–1.28), LBP (HR = 1.06, 95% CIs = 1.03–1.10), NP (HR = 1.12, 95% CIs = 1.00–1.24), and gout (HR = 1.34, 95% CIs = 1.26–1.42) but not with OA (Supplementary Table 14).

The hPDI-related metabolic signature showed a stronger inverse association with total MSDs than hPDI. Compared with Q1, individuals in Q5 had a significantly lower risk (HR = 0.95, 95% CIs = 0.90–0.99; per-SD: HR = 0.98, 95% CIs = 0.96–1.00; Table 2). Regarding specific MSDs, the hPDI-related metabolic signature was associated with a decreased risk of RA (HR = 0.93, 95% CIs = 0.87–0.99), OA (HR = 0.97, 95% CIs = 0.95–0.99), LBP (HR = 0.95, 95% CIs = 0.92–0.98), and gout (HR = 0.79, 95% CIs = 0.75–0.83) but not with NP (Supplementary Table 14).

### Mediation analysis

**Figure 2:**
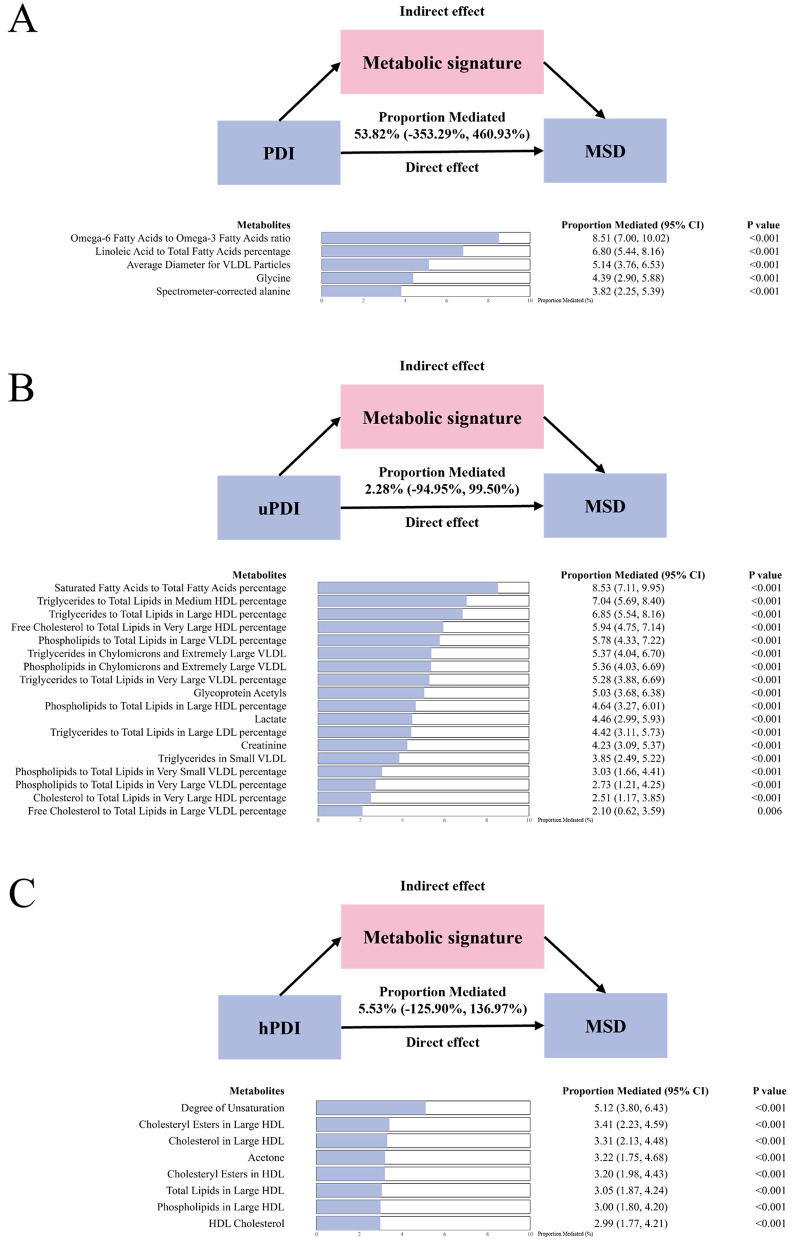
**(A)** Association of the PDI with total MSDs mediated by metabolic signature and metabolites. **(B)** Association of the uPDI with total MSDs mediated by metabolic signature and metabolites. **(C)** Association of the hPDI with total MSDs mediated by metabolic signature and metabolites. PDI, plant-based diet index; uPDI, unhealthy plant-based diet index; MSDs, musculoskeletal disorders; HDL, high-density lipoprotein; VLDL, very low-density lipoprotein.

Although none of the three PDIs-related metabolic signatures exerted a significant mediating effect in the PDIs-MSDs associations (all *P* > 0.05), several individual metabolites were identified as important mediators. In the PDI-total MSDs analysis, key mediators included omega-6/omega-3 fatty acid ratio, LA percentage, very low-density lipoprotein (VLDL) average diameter, glycine, and spectrometer-corrected alanine, each explaining 3.82%-8.51% of the association (Figure 2A). In the uPDI-total MSDs analysis, 18 metabolites explained 2.10%−8.53% of the association, including SFAs to total FAs percentage, triglyceride-related metabolites free cholesterol-related metabolites, and phospholipid-related metabolites (Figure 2B). In the hPDI-total MSDs analysis, eight metabolites explained 2.99%−5.12% of the association, including degree of unsaturation, cholesteryl esters and cholesterol in large HDL (Figure 2C). Results for each specific MSDs are shown in Supplementary Figures 3–7.

### PDIs, metabolic signatures, conventional lifestyle risk, and MSDs incidence

Compared to individuals with low conventional lifestyle score, those with high score had a significantly increased risk of MSDs (Supplementary Table 15). In stratified analysis, the associations of three PDIs, their related metabolic signatures, and the risk of MSDs remained consistent across strata. In joint analysis, individuals with high conventional lifestyle risk and unfavorable dietary pattern (reflected by low PDI, high uPDI, or low hPDI) showed the highest risk of total MSDs compared to their reference counterparts (those with low lifestyle risk and favorable dietary pattern). A similar yet more pronounced association was observed for the related metabolic signatures (Table 3). No multiplicative or additive interaction was observed. Results for each specific MSDs are shown in Supplementary Table 16.

**Table 3:** Joint effect, stratified analysis and interaction between PDIs, metabolic signatures and conventional lifestyle factors on the risk of total musculoskeletal disorders.

Exposure	Joint effect analysis				Additive interaction		Stratified analysis				Multiplicative interaction	
	Conventional lifestyle factors	Case	Person year	HR (95% CI)	Estimate (95% CI)	*P for interaction*	Conventional lifestyle factors	Case	Person year	HR (95% CI)^*^	HR (95% CI)	*P for interaction*
PDI												
High	Low	7,531	287,339.78	Reference	RERI −0.05 (−0.10 to 0.00)	0.050	Low	15,912	589,582.95	0.96 (0.93–0.99)	0.96 (0.91–1.00)	0.081
Low	Low	8,381	302,243.17	1.04 (1.01–1.07)	AP −0.04 (−0.08 to 0.00)	0.050						
High	High	6,913	209,968.21	1.20 (1.16–1.24)			High	15,185	459,577.32	1.00 (0.97–1.04)		
Low	High	8,272	249,609.11	1.19 (1.15–1.23)	S 0.85 (0.65–1.05)	0.803						
uPDI												
Low	Low	6,198	226,366.03	Reference	RERI 0.03 (−0.02 to 0.08)	0.237	Low	15,912	589,582.95	1.01 (0.98–1.04)	1.02 (0.98–1.07)	0.273
High	Low	9,714	363,216.91	1.01 (0.98–1.04)	AP 0.03 (−0.02 to 0.07)	0.240						
Low	High	5,405	163,534.31	1.15 (1.11–1.19)			High	15,185	459,577.32	1.04 (1.00–1.07)		
High	High	9,780	296,043.01	1.19 (1.15–1.23)	S 1.19 (0.87–1.64)	0.330						
hPDI												
High	Low	11,455	423,609.92	Reference	RERI −0.03 (−0.08 to 0.03)	0.617	Low	15,912	589,582.95	1.01 (0.98–1.05)	0.98 (0.93–1.02)	0.967
Low	Low	4,457	165,973.02	1.01 (0.98–1.05)	AP −0.02 (−0.07 to 0.02)	0.892						
High	High	10,847	325,356.99	1.18 (1.14–1.21)			High	15,185	459,577.32	0.99 (0.95–1.02)		
Low	High	4,338	134,220.32	1.16 (1.12–1.20)	S 0.86 (0.63–1.16)	0.734						
PDI-related metabolic signature												
Favorable	Low	4,100	160,187.49	Reference	RERI 0.02 (−0.04 to 0.09)	0.799	Low	7,872	282,502.43	0.93 (0.89–0.97)	1.03 (0.97–1.10)	0.548
Unfavorable	Low	3,772	122,314.94	1.08 (1.03–1.13)	AP 0.02 (−0.04 to 0.08)	0.744						
Favorable	High	3,398	109,007.05	1.17 (1.12–1.23)			High	7,622	225,112.30	0.95 (0.91–1.00)		
Unfavorable	High	4,224	116,105.25	1.23 (1.17–1.28)	S 1.41 (0.47–4.22)	0.823						
uPDI-related metabolic signature												
Unfavorable	Low	4,789	168,139.89	Reference	RERI 0.08 (−0.01 to 0.15)	0.063	Low	7,872	282,502.43	1.02 (0.97–1.06)	1.06 (0.99–1.13)	0.084
Favorable	Low	3,083	114,362.54	1.03 (0.98–1.07)	AP 0.06 (−0.01 to 0.12)	0.093						
Unfavorable	High	3,089	92,439.23	1.11 (1.06–1.16)			High	7,622	225,112.30	1.11 (1.06–1.16)		
Favorable	High	4,533	132,673.06	1.22 (1.17–1.28)	S 1.50 (0.97–2.30)	0.134						
hPDI-related metabolic signature												
Favorable	Low	4,534	167,202.55	Reference	RERI 0.00 (−0.07 to 0.07)	1.000	Low	7,872	282,502.43	0.92 (0.88–0.96)	1.01 (0.95–1.08)	0.945
Unfavorable	Low	3,338	115,299.88	1.09 (1.04–1.14)	AP 0.00 (−0.06 to 0.06)	1.000						
Favorable	High	3,068	93,390.40	1.15 (1.10–1.21)			High	7,622	225,112.30	0.92 (0.88–0.96)		
Unfavorable	High	4,554	131,721.90	1.24 (1.19–1.30)	S 1.02 (0.29–3.54)	0.474						

^*^HRs and 95 % CIs for stratified analysis were based on categorical variable, high vs. low.

Lifestyle factors including BMI, physical activity, smoking status, alcohol consumption and sleep duration (range from 0 to 5, low = 0–2 factors, high = 3–5 factors); models are adjusted for sex, age, TDI, education, energy intake, multivitamin use and history of treatments (antihyperlipidemic drugs, antihypertensive and antidiabetic drugs). PDI, plant-based diet index; uPDI, unhealthy plant-based diet index; hPDI, healthy plant-based diet index; HR, hazard ratio; 95% CI, 95% confidence interval; TDI, Townsend deprivation index; BMI, body mass index; RERI, relative excess risk due to interaction; AP, attributable proportion due to interaction; S, the synergy index.

### PDIs, metabolic signatures, genetic risk, and MSDs incidence

Compared to individuals with low genetic susceptibility (low PRS), those with high PRS had a significantly elevated risk of MSDs (Supplementary Table 17). In stratified analysis, the associations of three PDIs, their related metabolic signatures, and the risk of MSDs remained consistent across different metaPRS strata. In joint analysis, individuals with high genetic susceptibility and unfavorable dietary pattern showed the highest risk of total MSDs compared to their reference counterparts. A comparable yet more pronounced association was observed for the related metabolic signatures (Table 4). No multiplicative or additive interaction was observed. Results for each specific MSDs are shown in Supplementary Table 18.

**Table 4:** Joint effect, stratified analysis and interaction between PDIs, metabolic signatures and PRS on the risk of total musculoskeletal disorders.

Exposure	Joint effect analysis				Additive interaction		Stratified analysis				Multiplicative interaction	
	PRS	Case	Person year	HR (95% CI)	Estimate (95% CI)	*P for interaction*	PRS	Case	Person year	HR (95% CI)^*^	HR (95% CI)	*P for interaction*
PDI												
High	Low	5,897	212,682.80	Reference	RERI −0.03 (−0.09 to 0.02)	0.284	Low	12,706	447,070.04	0.98 (0.94–1.01)	0.96 (0.91–1.00)	0.232
Low	Low	6,809	234,387.24	1.03 (0.99–1.06)	AP −0.03 (−0.08 to 0.02)	0.240						
High	High	6,289	209,150.41	1.09 (1.05–1.13)			High	13,438	442,051.20	1.00 (0.97–1.04)		
Low	High	7,149	232,900.79	1.08 (1.04–1.12)	S 0.72 (0.44–1.17)	0.130						
uPDI												
Low	Low	4,778	168,085.07	Reference	RERI 0.00 (−0.06 to 0.05)	1.000	Low	12,706	447,070.04	1.02 (0.98–1.06)	1.02 (0.98–1.07)	0.862
High	Low	7,928	278,984.96	1.02 (0.98–1.06)	AP 0.00 (−0.05 to 0.05)	1.000						
Low	High	5,036	164,954.60	1.07 (1.03–1.12)			High	13,438	442,051.20	1.02 (0.98–1.06)		
High	High	8,402	277,096.60	1.09 (1.05–1.13)	S 0.97 (0.53–1.75)	0.923						
hPDI												
High	Low	9,038	319,592.05	Reference	RERI −0.05 (−0.11 to 0.01)	0.617	Low	12,706	447,070.04	0.96 (0.92–1.00)	0.96 (0.90–1.01)	0.119
Low	Low	3,668	127,477.98	1.03 (0.99–1.08)	AP −0.04 (−0.10 to 0.01)	0.892						
High	High	9,662	315,242.25	1.08 (1.05–1.12)			High	13,438	442,051.20	1.01 (0.97–1.06)		
Low	High	3,776	126,808.94	1.07 (1.03–1.12)	S 0.61 (0.33–1.13)	0.734						
PDI-related metabolic signature												
Favorable	Low	3,018	114,435.12	Reference	RERI 0.00 (−0.07 to 0.07)	1.000	Low	6,289	215,284.40	0.95 (0.90–1.00)	1.00 (0.94–1.08)	0.41
Unfavorable	Low	3,271	100,849.28	1.06 (1.01–1.12)	AP 0.00 (−0.07 to 0.07)	1.000						
Favorable	High	3,256	112,202.08	1.09 (1.04–1.15)			High	6,708	212,744.49	0.96 (0.92–1.01)		
Unfavorable	High	3,452	100,542.41	1.13 (1.07–1.18)	S 1.05 (0.12–9.40)	0.983						
uPDI-related metabolic signature												
Unfavorable	Low	3,255	112,924.03	Reference	RERI −0.02 (−0.09 to 0.06)	0.601	Low	6,289	215,284.40	1.03 (0.98–1.09)	0.98 (0.92–1.05)	0.363
Favorable	Low	3,034	102,360.37	1.02 (0.97–1.08)	AP −0.01 (−0.08 to 0.05)	0.762						
Unfavorable	High	3,409	108,145.86	1.09 (1.04–1.15)			High	6,708	212,744.49	0.99 (0.94–1.04)		
Favorable	High	3,299	104,598.63	1.08 (1.03–1.14)	S 0.87 (0.48–1.58)	0.643						
hPDI-related metabolic signature												
Favorable	Low	3,067	111,878.02	Reference	RERI 0.05 (−0.02 to 0.12)	1.000	Low	6,289	215,284.40	0.92 (0.87–0.97)	1.05 (0.98–1.13)	0.617
Unfavorable	Low	3,222	103,406.38	1.09 (1.03–1.14)	AP 0.05 (−0.02 to 0.12)	1.000						
Favorable	High	3,324	108,054.15	1.12 (1.06–1.17)			High	6,708	212,744.49	1 (0.95–1.05)		
Unfavorable	High	3,384	104,690.34	1.12 (1.07–1.18)	S 0.87 (0.48–1.58)	0.474						

^*^HRs and 95 % CIs for stratified analysis were based on categorical variable, high vs. low.

Cox regression model was adjusted for sex, age, array, PCA1-PCA10, TDI, education, physical activity, BMI, smoking status, alcohol consumption, sleep duration, and energy intake, multivitamin use and history of treatments (antihyperlipidemic drugs, antihypertensive and antidiabetic drugs). PDI, plant-based diet index; uPDI, unhealthy plant-based diet index; PRS, polygenic risk score; HR, hazard ratio; 95% CI, 95% confidence interval; TDI, Townsend deprivation index; BMI, body mass index; RERI, relative excess risk due to interaction; AP, attributable proportion due to interaction; S, the synergy index.

### Sensitivity analysis

To provide accurate dietary recommendations, additional analyses were conducted to explore the associations between 17 individual food groups and the risk of MSDs. Higher consumption of whole grains (HR = 0.90, 95% CIs = 0.88–0.96), vegetables (HR = 0.96, 95% CIs = 0.92–1.00), and dairy products (HR = 0.94, 95% CIs = 0.91–0.98) were significantly associated with a reduced risk of total MSDs. Additionally, higher intake of fruits and seafood was also linked to a lower risk of specific MSDs (Supplementary Table 19).

The associations of PDIs with the risk of incident total and specific MSDs remained largely consistent in sensitivity analysis where the cases occurring within the initial 1 year of follow-up were excluded, individuals with fewer than two dietary assessments were excluded, and competing risk models accounting for mortality were applied (Supplementary Table 20).

## Discussion

This large-scale, prospective cohort study investigated the associations between plant-based diets, their corresponding metabolic signatures, and the risk of MSDs, while taking into account conventional risk factors and genetic susceptibility. Our principal findings reveal that: (1) plant-based diets is associated with a lower risk of MSDs, most likely driven by hPDI, while uPDI increases such a risk; (2) the magnitude of effects become stronger when constructing metabolic signatures to better reflect biological responses; (3) the inverse associations of favorable dietary patterns and their corresponding metabolic signatures with MSDs risk remained consistent across strata of genetic susceptibility and conventional lifestyle risk profile; and (4) key metabolites related to fatty acid composition and lipoprotein metabolism, though not the overall signatures, were identified as significant mediators, illuminating potential mechanistic pathways linking diet to MSDs development.

Previous epidemiological studies have established preliminary links between plant-based diets and specific MSDs. For example, a randomized crossover trial (*N* = 32) demonstrated that a calorie-unrestricted plant-based elimination diet markedly lowered disease activity and reduced number of swollen joints in RA patients (42). Similarly, a 6-week randomized open-label study (*N* = 37) reported improvements in physical function and pain among OA patients following a whole-foods plant-based diet (43). Additionally, two prospective cohort studies (*N* = 4,903 and 9,032, respectively) in East Asian populations also found that vegetarian diets were associated with a lower risk of gout (44). Despite these valuable insights, the existing body of evidence is characterized by relatively small sample sizes and a narrow focus on individual MSDs, often without differentiating between the quality of plant-based food sources. Our study extends this prior evidence in several critical dimensions. First, we comprehensively examined the roles of PDIs, further refined into hPDI and uPDI, in the risk of MSDs, offering broader insights into the potential for co-prevention and co-management of major MSDs. Second, by leveraging a large-scale cohort of 107,983 individuals, we substantially augmented the sample size from previous studies, which typically included a few thousand participants at most. This vast increase in scale significantly improves statistical power and helps identify effect sizes that previous studies did not have the opportunity for. While our findings indicate statistically significant associations, the modest effect sizes warrant cautious interpretation of their clinical and public health implications.

Another pivotal contribution of our study is the development of objective metabolic signatures to capture the systemic biological responses to plant-based diets. Crucially, these metabolite scores showed stronger and more robust associations with MSDs risk than the original questionnaire-based dietary indices. For instance, while the protective association of hPDI with the risk of MSDs was only marginally significant using traditional epidemiological approaches, its corresponding metabolic signature revealed a substantially stronger protective effect. This pattern of effect amplification was consistently observed for the overall PDI and uPDI signatures as well. While it could be posited that these signatures merely represent a transformed reflection of the original dietary exposure, our mutually adjusted models (Model 3) demonstrated that the signatures remained significantly associated with MSDs risk independently of the corresponding dietary indices, indicating that they capture additional variance in disease risk not explained by self-reported dietary intake alone. This suggests that metabolomic profiles more accurately capture the biological implementation of diet, potentially mitigating the measurement error inherent in self-reported data and providing a more integrated reflection of an individual's metabolic state. These findings significantly strengthen causal plausibility by directly linking dietary patterns to downstream disease risk through measurable metabolic perturbations. Furthermore, by pinpointing specific biological pathways—such as those involving fatty acid composition and lipoprotein metabolism—these signatures move beyond association to offer mechanistic insights. This approach identifies potential biomarkers and targets for nutritional interventions, providing a translational bridge that traditional dietary assessments alone cannot establish.

Although the overall metabolic signatures did not show significant mediation, the identification of individual metabolites that partially explain the diet-MSDs relationship offers valuable mechanistic insights. The protective association of plant-based diets was characterized by favorable lipid profiles, with LA emerging as the most responsive metabolite in the PDI-related signature. LA contributes to musculoskeletal health through both its direct anti-inflammatory properties and its ability to reduce visceral adipose tissue, thereby mitigating systemic inflammation and creating a favorable bone microenvironment (45). Similarly, the hPDI-related signature showed greater FA unsaturation and enrichment of cholesteryl esters and cholesterol in large HDL, suggesting enhanced lipid fluidity and efficient reverse cholesterol transport (46) that support bone and cartilage homeostasis (47). In contrast, the uPDI-related signature was marked by elevated SFAs and TGs, which impair the formation of bone-forming osteoblasts (48) and increase the expression of inflammatory genes (TNFα, IL-4, IL-17, and P53) (49), potentially exacerbating inflammation and oxidative stress (50). Collectively, these findings identify lipid metabolism as a key pathway linking plant-based dietary quality to musculoskeletal health, with high unsaturation profiles predominantly mediating the protective effects.

The associations between plant-based diets and risk of MSDs remained consistent across all strata of lifestyle factors and genetic susceptibility. The absence of significant lifestyle-diet or gene-diet interactions indicates that the benefits of plant-based diets extend broadly across the whole population, independent of their baseline risk profile. These findings underscore the population-wide applicability of plant-based dietary recommendations for MSDs prevention and highlight their potential as equitable and accessible public health interventions.

For the first time, our analysis revealed nuanced associations between plant-based diets and specific MSDs, reflecting the distinct pathological mechanisms underlying each condition. For inflammatory conditions such as RA, LBP, and NP, uPDI was the primary driver of increased risk. This pattern, characterized by high levels of SFAs and TGs, is known to elevate circulating free fatty acids, thereby activating inflammatory signaling cascades (e.g., TNF-α, IL-6) that amplify disease pathogenesis (51). In contrast, for degenerative conditions like OA, PDI and hPDI were significantly associated with reduced risk. This protective effect aligns with the capacity of these diets to supply key nutrients that sustain cartilage matrix integrity and regulate chondrocyte metabolism, thereby slowing degenerative progression (52). For the metabolic conditions of gout, all three dietary indices, particularly their corresponding metabolic signatures, showed pronounced associations. This is consistent with the central role of uric acid metabolism in gout. Plant-based diets mitigate hyperuricemic burden by limiting dietary purine intake, promoting uric acid excretion, and consequently reducing the deposition of urate crystals that trigger gout flares (15).

Several limitations should be considered. First, dietary data were collected via 24-h recalls, which may not fully reflect long-term habitual intake and could introduce non-differential misclassification that attenuates observed associations, although sensitivity analysis verified the robustness of results. Second, the demographic characteristics of UKB limit generalizability to non-European populations, where dietary patterns and genetic architectures differ to a large extent. Meanwhile, our analyses were restricted to participants with complete dietary and metabolomic data. Combined with the inherent healthy volunteer bias in the UK Biobank cohort, this may lead to selection bias and further reduce the generalizability of our results. Third, dietary intake and metabolomic mediators were assessed concurrently at baseline. While our prospective design ensures exposure and mediators precede incident MSDs and rules out reverse causality, it cannot confirm the ideal exposure-to-mediator temporal order. Additionally, baseline metabolic profiling was cross-sectional, precluding assessment of dynamic changes in metabolic signatures over follow-up and thus limiting our understanding of how metabolic traits evolve alongside MSDs risk. Moreover, our MSDs outcome aggregates multiple heterogeneous musculoskeletal conditions with distinct etiologies and pathological mechanisms, which may introduce outcome heterogeneity and obscure disease-specific associations. Finally, residual confounding from unmeasured factors (e.g., nonsteroidal anti-inflammatory drug use, autoimmune diseases) cannot be fully controlled even after adjusting for key covariates.

## Conclusions

This large-scale prospective study provides robust evidence that the quality of a plant-based diets is independently associated with the risk of developing MSDs. Moving beyond simple dietary questionnaires, we established objective metabolic signatures that more powerfully capture the biological link between diet and disease. These signatures, along with the identified mediating metabolites, illuminate lipid metabolism—particularly a shift toward beneficial unsaturated FAs—as a central mechanistic pathway and offer new targets for future interventions. Our results underscore the importance of promoting high-quality, healthy plant-based diets rich in whole grains, vegetables, and fruits as a foundational strategy for the primary prevention of MSDs across the general population.

## Data Availability

The original contributions presented in the study are included in the article/Supplementary material, further inquiries can be directed to the corresponding authors.
