# Supplementary material for: Plant-based diet, metabolic signature, genetic susceptibility, and risk of musculoskeletal disorders: a large-scale population-based prospective cohort study
Source: Front Public Health. 2026 Jun 29;14:1803631. doi: 10.3389/fpubh.2026.1803631 (PMC13357934; doi:10.3389/fpubh.2026.1803631)
Supplement: Supplementary file 1 [file Data_Sheet_1.docx]

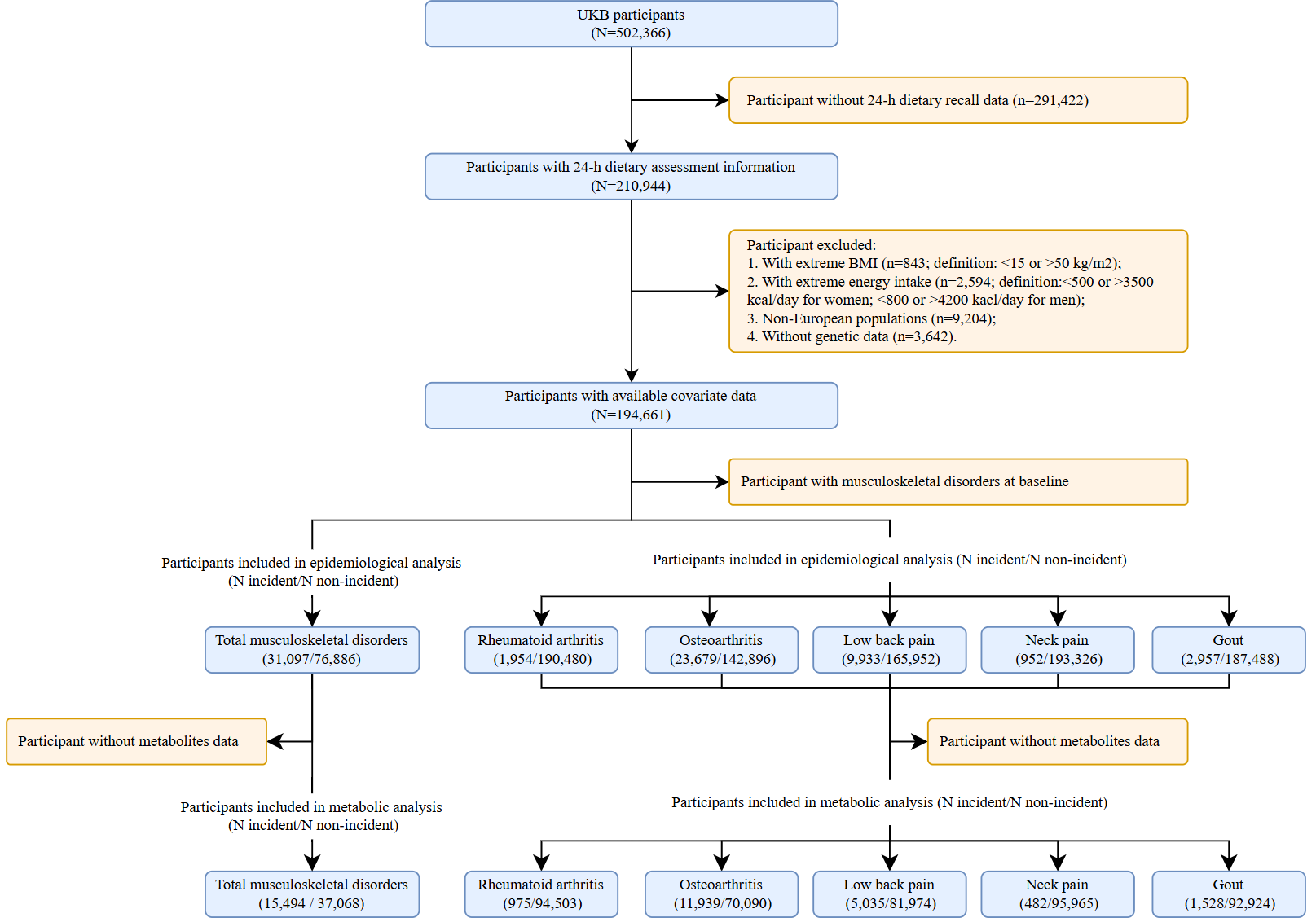


**Supplementary Figure 1.** Flow diagram of participant inclusion and exclusion among participants from the UK Biobank. Abbreviations: BMI, body mass index.


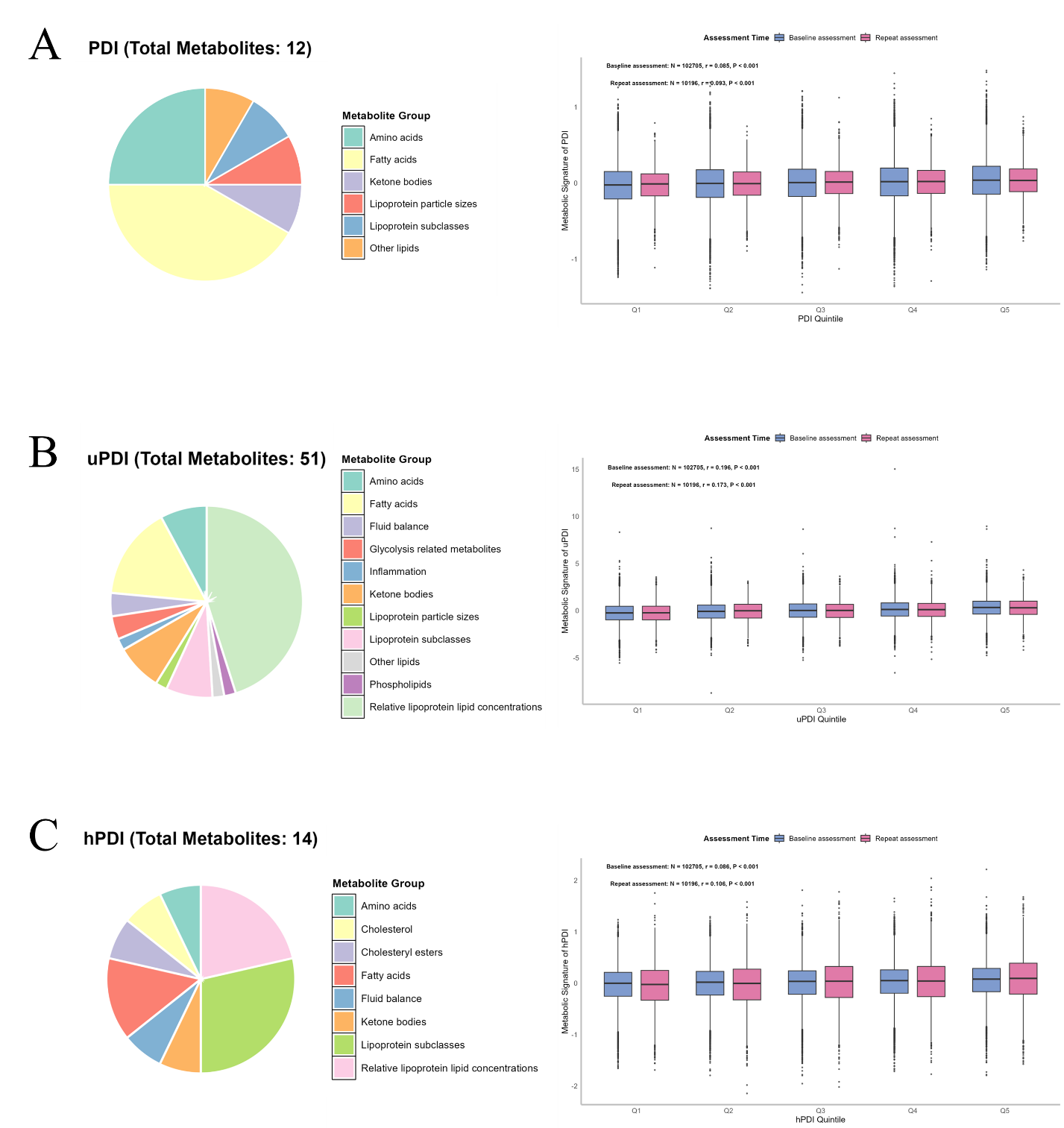


**Supplementary Figure 2. (A)** PDI-related metabolic signature consists of 12 metabolites identified by regressing healthy lifestyle score on total 251 metabolites using elastic net regression and the correlations between PDI and the metabolic signature at baseline and repeated assessment. **(B)** uPDI-related metabolic signature consists of 51 metabolites identified by regressing healthy lifestyle score on total 251 metabolites using elastic net regression and the correlations between uPDI and the metabolic signature at baseline and repeated assessment. **(C)** hPDI-related metabolic signature consists of 14 metabolites identified by regressing healthy lifestyle score on total 251 metabolites using elastic net regression and the correlations between hPDI and the metabolic signature at baseline and repeated assessment. Abbreviations: PDI, plant-based diet index; uPDI, unhealthy plant-based diet index; hPDI, healthy plant-based diet index.


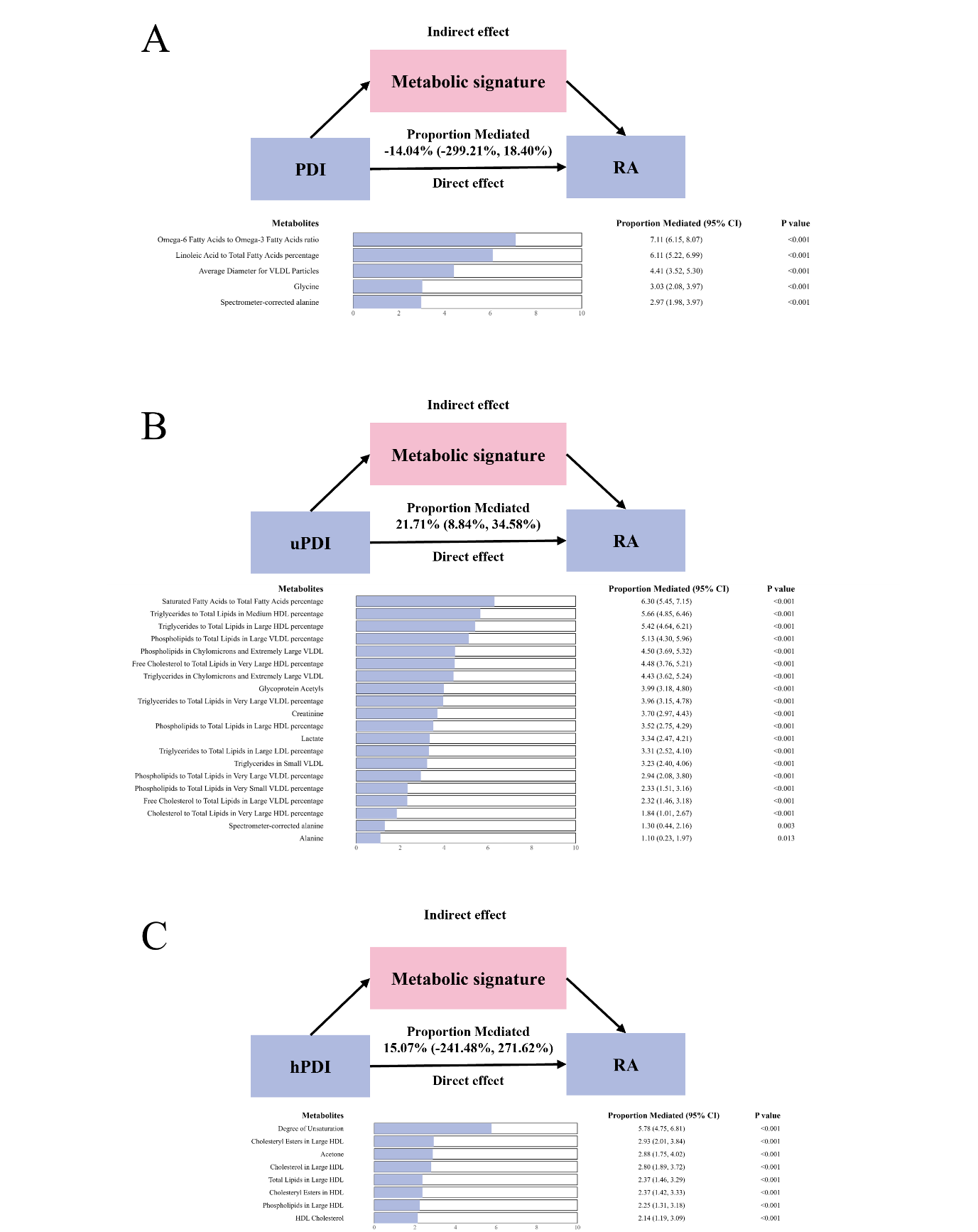


**Supplementary Figure 3. (A)** Association of the PDI with RA mediated by metabolic signature and metabolites. **(B)** Association of the uPDI with RA mediated by metabolic signature and metabolites. **(C)** Association of the hPDI with RA mediated by metabolic signature and metabolites. Abbreviations: PDI, plant-based diet index; uPDI, unhealthy plant-based diet index; hPDI, healthy plant-based diet index; RA, rheumatoid arthritis.


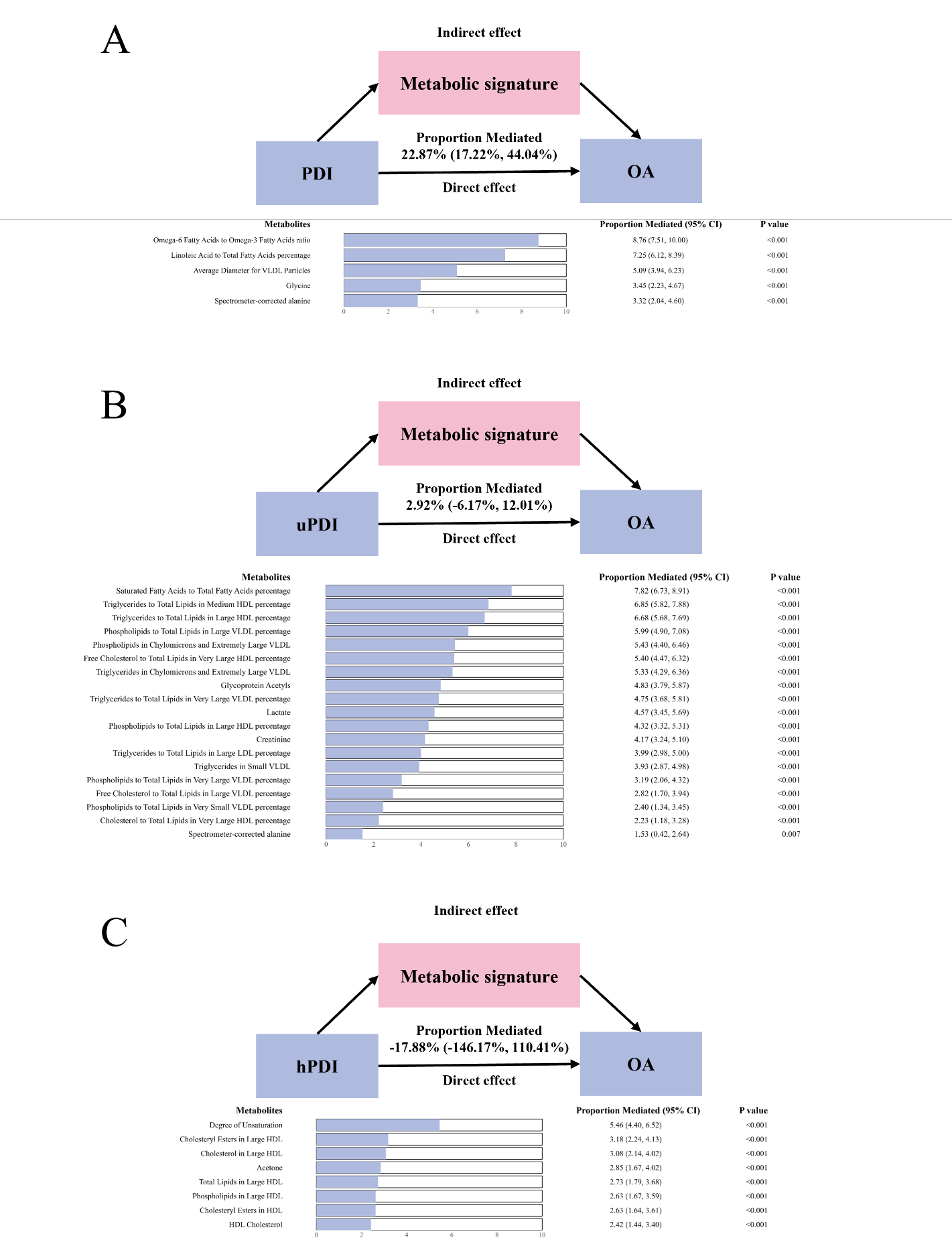


**Supplementary Figure 4. (A)** Association of the PDI with OA mediated by metabolic signature and metabolites. **(B)** Association of the uPDI with OA mediated by metabolic signature and metabolites. **(C)** Association of the hPDI with OA mediated by metabolic signature and metabolites. Abbreviations: PDI, plant-based diet index; uPDI, unhealthy plant-based diet index; hPDI, healthy plant-based diet index; OA, osteoarthritis.


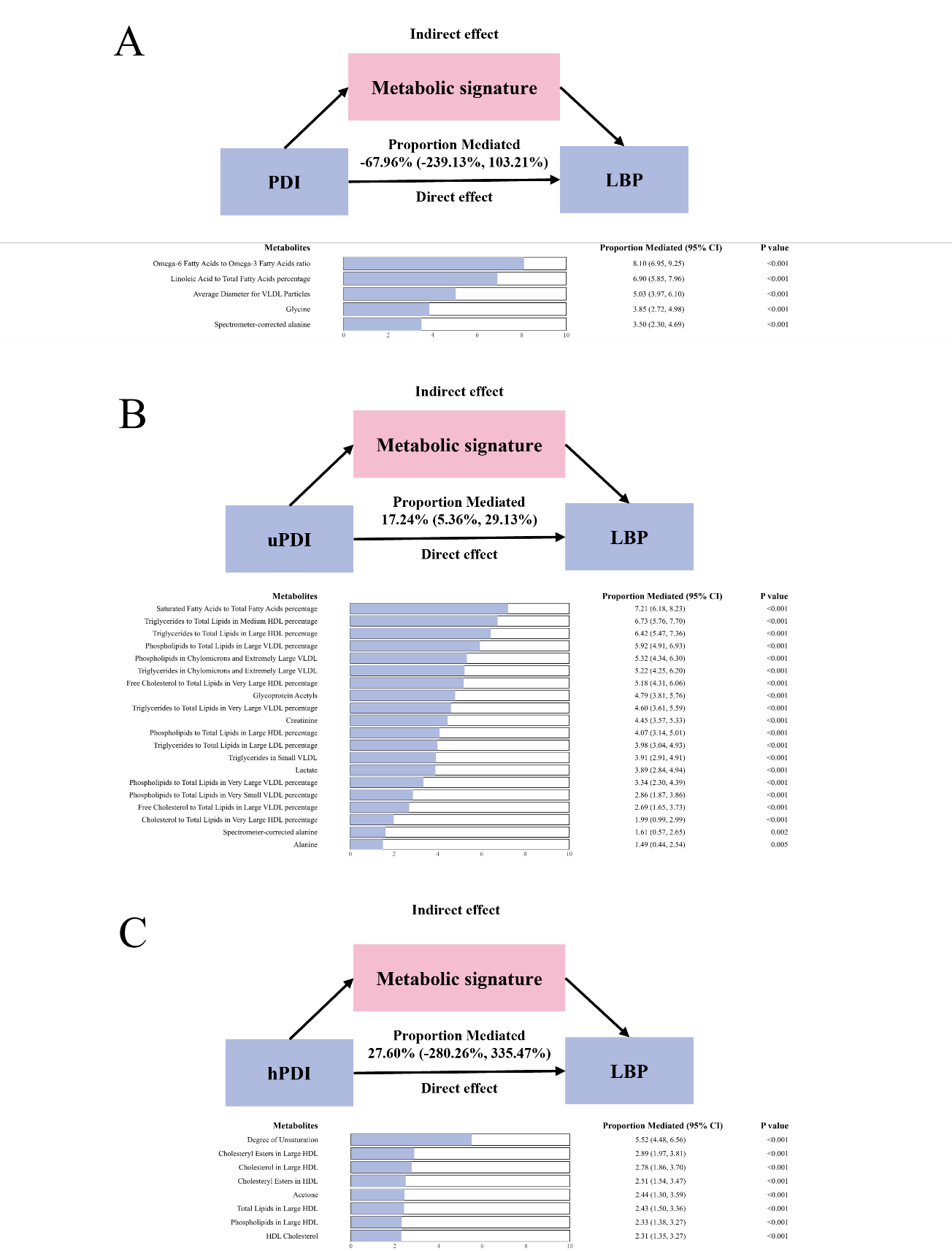


**Supplementary Figure 5. (A)** Association of the PDI with LBP mediated by metabolic signature and metabolites. **(B)** Association of the uPDI with LBP mediated by metabolic signature and metabolites. **(C)** Association of the hPDI with LBP mediated by metabolic signature and metabolites. Abbreviations: PDI, plant-based diet index; uPDI, unhealthy plant-based diet index; hPDI, healthy plant-based diet index; LBP, low back pain.
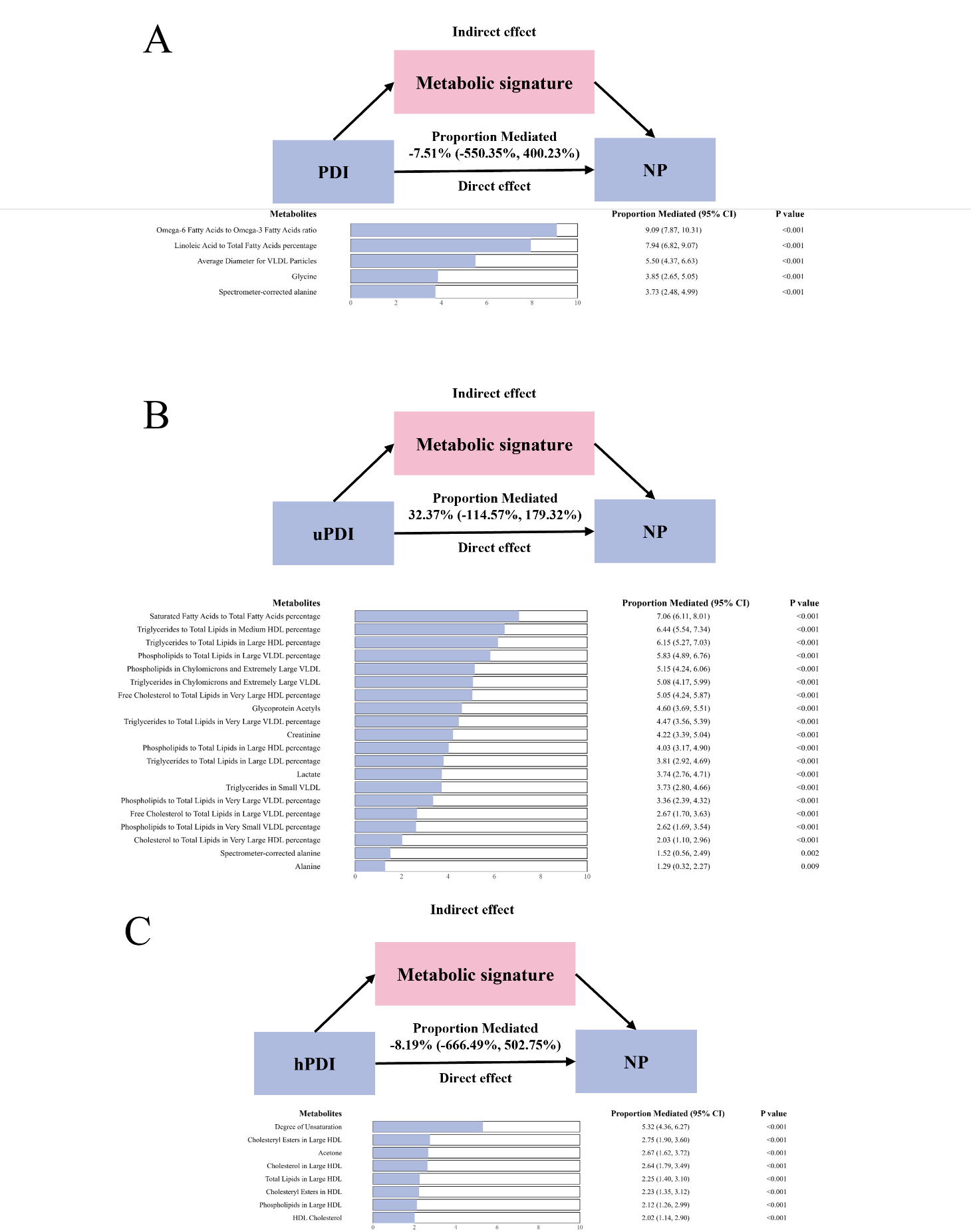


**Supplementary Figure 6. (A)** Association of the PDI with NP mediated by metabolic signature and metabolites. **(B)** Association of the uPDI with NP mediated by metabolic signature and metabolites. **(C)** Association of the hPDI with NP mediated by metabolic signature and metabolites. Abbreviations: PDI, plant-based diet index; uPDI, unhealthy plant-based diet index; hPDI, healthy plant-based diet index; NP, neck pain.


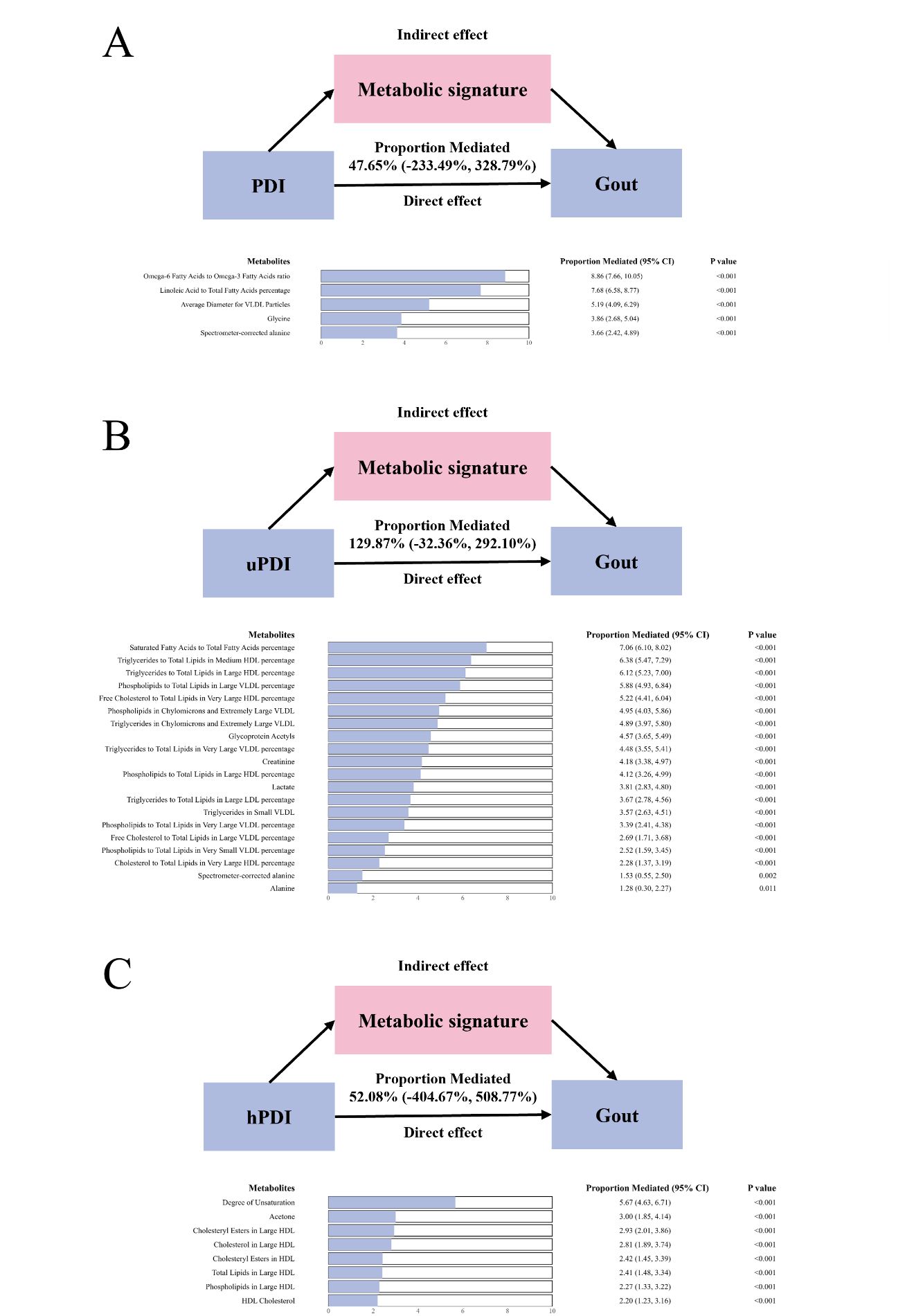


**Supplementary Figure 7. (A)** Association of the PDI with gout mediated by metabolic signature and metabolites. **(B)** Association of the uPDI with gout mediated by metabolic signature and metabolites. **(C)** Association of the hPDI with gout mediated by metabolic signature and metabolites. Abbreviations: PDI, plant-based diet index; uPDI, unhealthy plant-based diet index hPDI, healthy plant-based diet index.
